# Supplementary material for: TGF-β1 in plasma and cerebrospinal fluid can be used as a biological indicator of chronic pain in patients with osteoarthritis
Source: PLoS One. 2022 Jan 21;17(1):e0262074. doi: 10.1371/journal.pone.0262074 (PMC8782532; doi:10.1371/journal.pone.0262074)
Supplement: Attachment — Submitted filename: 2021-1026-Reply to the Reviewers comment.docx [file pone.0262074.s002.docx]

**Reviewers' comments:**

**Response:** We thank the reviewers for the positive words. As recommended by the reviewers, we have added the revision in the manuscript.

*Reviewer #1: I appreciate the authors’ efforts in revising their manuscript, which goes some way to address my original concerns. I suspect that my original comments (and those of the second reviewer where similar) might have been incompletely understood by the authors and I attempt to explain my residual concerns more clearly below.

What was the lead question and recall timeframe for the pain VAS? (?p6) I note also that this point was raised by the second reviewer, who made additional pertinent points. Having read the authors’ response, I think that I might understand the source of apparent confusion and discrepancies, but this is not yet clear to the general reader of the manuscript. Am I correct to think that participants were recruited on the basis of clinical evidence of chronic pain (undergoing arthroplasty), but that the pain VAS used in analyses asked about `recent pain’. As in my original comments, please give the exact text and anchors for the VAS used. I expect this was not in English language, and so the exact text in the original language, plus a verbatim translation might suffice. It makes a very big difference what time-frame defined `recent’ if the original text used the word `recent’. Does `recent’ mean since admission, over the past few minutes while you have not been standing, or over the past few months? I suspect from the results that participants rated their `current’ pain whilst not mobilising or standing. Our experience also is that patients listed for arthroplasty might not have severe pain at rest, but as soon as they try to do something, then their pain becomes severe. Chronic pain (e.g. as measured by WOMAC or clinically used VAS scores) typically refers to pain over the past week; either average pain, or worst pain, thereby reflecting the intermittent nature often of OA pain. On reflection, I can understand that `current pain’ might be the most appropriate index here, because molecules in CSF might change over short time periods, and the authors might wish to measure the pain experienced at a time point closest to biosample collection.*

**Response:** Thank you for your comments. Correctly, all patients were asked to evaluate their current pain intensity using the Visual Analogue Scale (VAS, ranged from 0-10, no moving or standing), based on their native language Mandarin or Taiwanese a day before the operation and biosample collection. All of these were revised in method (Page 6, last paragraph) and results (Page 8, last paragraph).

*However, if this is the case, they might wish to speculate as to how relevant this might be to clinical pain. At least, they should explain the apparent inconsistency commented on by both reviewers that people with very low pain scores were undergoing joint surgery.*

**Response:** Thank you for your comments. And, as you recognized, our data support that patients with end-stage OA experienced with chronic pain (> 1000 days, Table 1). In addition, our data also revealed that some patients with chronic OA do not feel obvious pain when resting (VAS ≤ 2, in OA with very mild pain group). And this patients (OA with very mild pain) at rest had higher CSF TGF-β1 than those OA patients with pain. Thus, we hypothesize that the levels of TGF-β1 in CSF in humans may modulate pain in OA subjects. All of these were revised in result (Page 8, last paragraph; Page 9, last paragraph) and discussion (Page 20, line 328-330).

*Also I note that reviewer 2, like myself, commented on the GU comparison group was selected as “having no pain”; so am I correct to understand that they had no `current pain on admission to hospital’ but that they might have had chronic pain prior to admission? This again should be clarified, and might be part of the same explanation as the clarification of what the VAS score represents.*

**Response:** Thank you for your comments. We apology that we did not described the definition and condition of the GU-control group clearly. In this study, patients with GU diseases without obvious current pain at rest (VAS score 0-1, mean VAS score is 0.3 ± 0.6 in GU control patients, Table 1) before surgery were included as a comparison (control) group. Furthermore, to access that they might have had chronic pain, we also checked the experience of pain of GU patients according to their recollection (GU control, mean duration of pain, 75.1 days in Table 1). All of these were revised in method (Page 6, lines 116-117) and result (Page 9).

*The authors have chosen to not `indicate that what was measured was immunoreactivity (e.g. BDNF-LI)’. I do not feels strongly about this, but the fact that ELISA measurements, especially competitive ELISAs, might measure factors other than the specific molecular species targeted, particularly when they are applied to biofluids in which the manufacturer and investigator have not validated them. I would still suggest that the authors provide data on validation in plasma and CSF, rathe than assume that these kits do what the manufacturers say they do when they sell them for profit.*

**Response:**

We agree that although the ELISA kit were validated to measure these molecules in human plasma and supernatants of cell culture. And the recombined human IL-8, TNF-α, BDNF and TGF-β1 diluted in buffer were used as a calibration. However, this is the first time that using of ELISA kits to detect these molecules in CSF, and the interpretation of CSF data should be more cautious. We have added and revised such description in method (Page 7, last paragraph; Page 8, 1^st^ paragraph) and limitations (Page 21).

*Please provide statistical evidence that log transformed data are normally distributed. If, as stated in the authors’ response, untransformed data were significantly positively skewed, then please use log transformed (or otherwise normalised) data for all parametric analyses, including between group comparisons. If log transformation converts non-normal data to normal data, then please only present analyses on log transformed data (e.g. in Table 2). In other words, only present the statistically appropriate analyses.*

**Response:** Thanks for the suggestion of statistics. Because of the log transformed data are still not normally distributed. For the biological molecules are often non-normality, thus, a non-parametric statistical test (Kruskal-Wallis test) was added to analysis the original data (Table 2). To determine the correlation between the VAS pain scores and the plasma and CSF molecule, the Spearman correlations with control of age, gender, body mass index and pain drug were performed (Table 3). All of these were added in method (Page 8, 2^nd^ paragraph), Table 2, Table 3 and results (Page 12, Page 15).

*I’m afraid that I don’t understand the data in the statement; ` The mean duration of pain was 75.1, 1,665, and 1,060 days with no pain, OA patients with very mild pain, and OA patients experiencing pain according to the recall of the patients, respectively (p < 0.0001).’ Are the authors really stating that participants were asked how long they had had pain to the nearest 0.1 days??? Given my above comments about variability of pain in OA, this seems meaningless if I am interpreting correctly. What is the method for this?*

**Response:** We apology that we did not clearly described the definition of this data. In this study, to confirm the situation of chronic pain, the duration of pain (days) was collected. Patients were asked to recall how long the pain or discomfort caused by the disease has persisted. All of these were added and revised in Table 1, method (Page 7, 1^st^ paragraph) and in result (Page 9, last paragraph).

*Reviewer #2: My concerns have been answered. The authors' findings are interesting although their significance is uncertain in view of conflicting evidence in thre literature on the roles of TGFbeta1 and BDNF.*

**Response:** Thank you for you insightful feedback and comments.
